# Supplementary material for: Continuous synthesis of high-entropy alloy nanoparticles by in-flight alloying of elemental metals
Source: Nat Commun. 2024 Feb 16;15:1450. doi: 10.1038/s41467-024-45731-z (PMC10873330; doi:10.1038/s41467-024-45731-z)
Supplement: Supplementary file 1 — Supplementary Information File [file 41467_2024_45731_MOESM1_ESM.pdf]

Supplementary Information

Continuous Synthesis of High-entropy Alloy Nanoparticles by In-flight Alloying of Elemental Metals

Keun Su Kim[1,2,3*], Martin Couillard[4], Ziqi Tang[3], Homin Shin[1], Daniel Poitras[5], Changjun Cheng[6], Olga Naboka[7], Dean Ruth[1], Mark Plunkett[1], Lixin Chen[6], Liliana Gaburici[1], Thomas Lacelle[1], Michel Nganbe[3], and Yu Zou[6]

[1]*Security and Disruptive Technologies Research Centre, National Research Council Canada, Ottawa, ON K1A 0R6, Canada*

[2]*Department of Mechanical and Industrial Engineering, University of Toronto, Toronto, ON M5S 3G8, Canada*

[3]*Department of Mechanical Engineering, University of Ottawa, Ottawa, ON, K1N 6N5, Canada*

[4]*Energy, Mining and Environment Research Centre, National Research Council Canada, Ottawa, ON K1A 0R6, Canada*

[5]*Advanced Electronics and Photonics Research Centre, National Research Council Canada, Ottawa, ON K1A 0R6, Canada*

[6]*Department of Materials Science and Engineering, University of Toronto, Toronto, ON M5S 3G8, Canada*

[7]*Construction Research Centre, National Research Council Canada, Ottawa, ON K1A 0R6, Canada*

[*]Corresponding author: Keun Su Kim; Tel: +1-613-998-5365; Fax: +1-613-991-2648

E-mail address: KeunSu.Kim@nrc-cnrc.gc.ca (K. S. Kim)

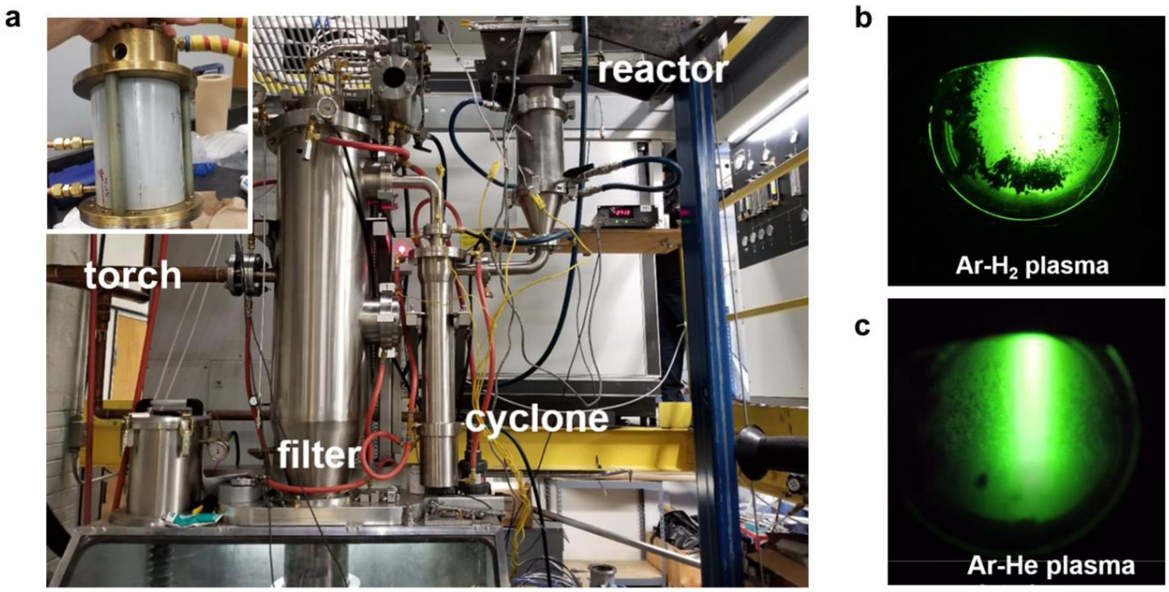

Supplementary Figure 1 | Photos of the processing system. a Photo of an inductively coupled plasma jet (ICPJ) process developed for the continuous synthesis of HEA NPs. The system consists of five major parts of an induction plasma torch, a reaction chamber (1-m long), a cyclone separator (ID: 0.034 m; OD: 0.097 m; length: 0.6 m), a filtration chamber (surface area = 20 × 50 cm, 2.8 μm pore size), and feedstock delivery (not shown here). **b** Photo of a plasma jet generated with Ar-H_2 (H_2: 8.3%). **c** Photo of a plasma jet generated with Ar-He (He: 77.4%).

Supplementary Table 1 | Summary of the mass balance and conversion efficiency in a typical ICPJ process.

Amount of powder fed	Running time	Powder collected			Productivity	Conversion efficiency
		reactor	cyclone	filter		
200 g	150 min	74 g	42 g	84 g	33.6 g h^{-1}	42 %

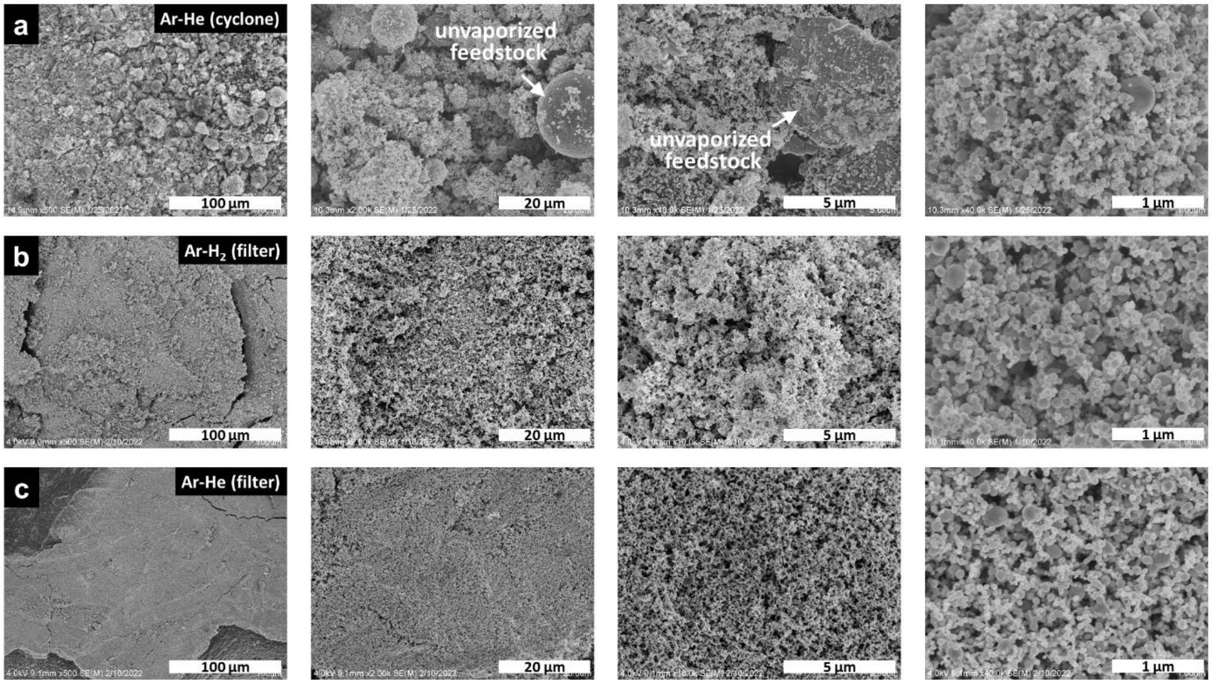

Supplementary Figure 2 | SEM analysis. SEM images of as-produced HEA NPs collected from **a** the cyclone separator and **b,c** the filter unit at different magnifications. Arrows indicate unvaporized feedstock separated by the cyclone.

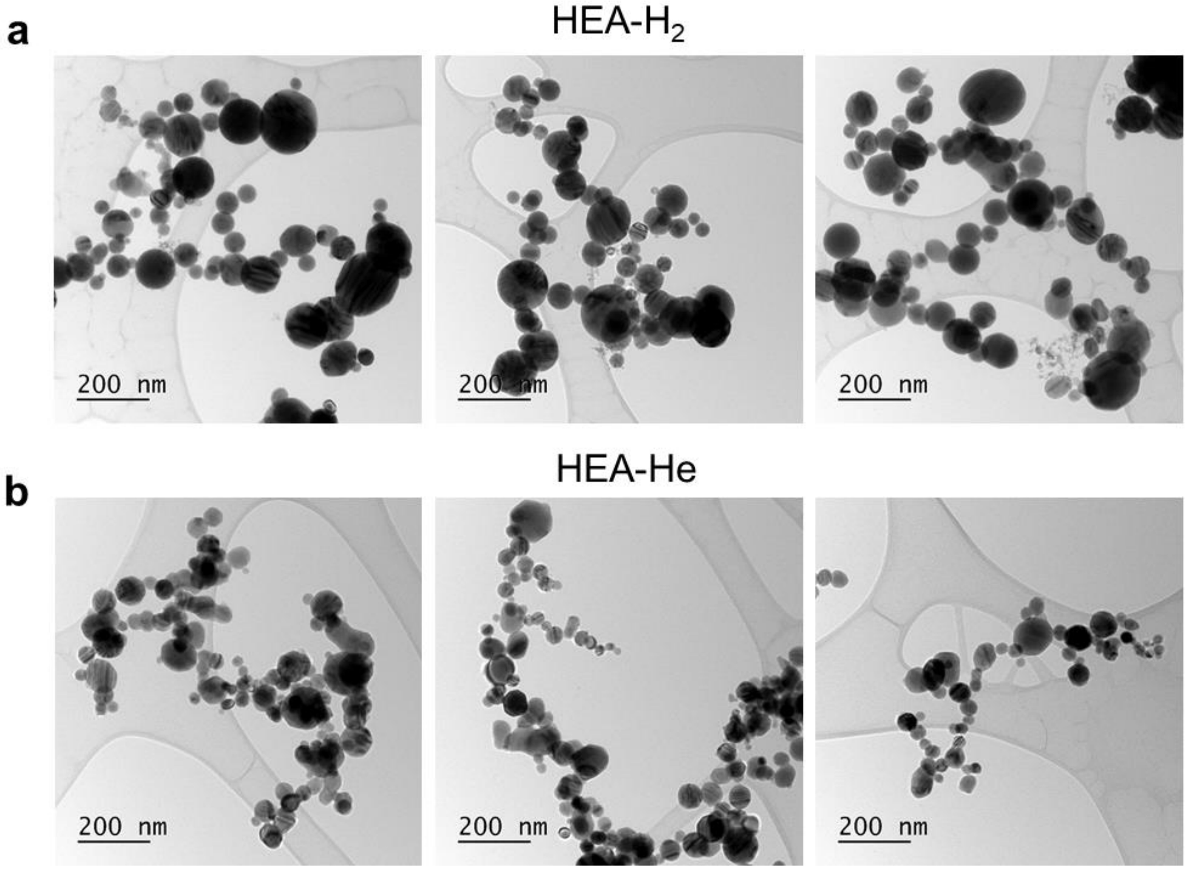

Supplementary Figure 3 | TEM analysis. TEM images of as-produced HEA NPs. **a** Sample produced with Ar-H_2 plasma gas (HEA-H_2, H_2: 8.3%). **b** Sample produced with Ar-He plasma gas (HEA-He, He: 77.4%).

Supplementary Table 2 | Interplanar spacing and lattice constants of CrFeCoNiMo HEA NPs synthesized with Ar-H_2 plasma gas.

hkl	$h^2 + k^2 + l^2$	*d* spacing (Å)	Lattice constant (Å)
111	1.732	2.0661	3.5786
200	2	1.7879	3.5759
220	2.828	1.2668	3.5830
			Average: 3.5792

Supplementary Table 3 | Interplanar spacing and lattice constants of CrFeCoNiMo HEA NPs synthesized with Ar-He plasma gas.

hkl	$h^2 + k^2 + l^2$	*d* spacing (Å)	Lattice constant (Å)
111	1.732	2.0661	3.5786
200	2	1.7912	3.5824
220	2.828	1.2659	3.5806
			Average: 3.5805

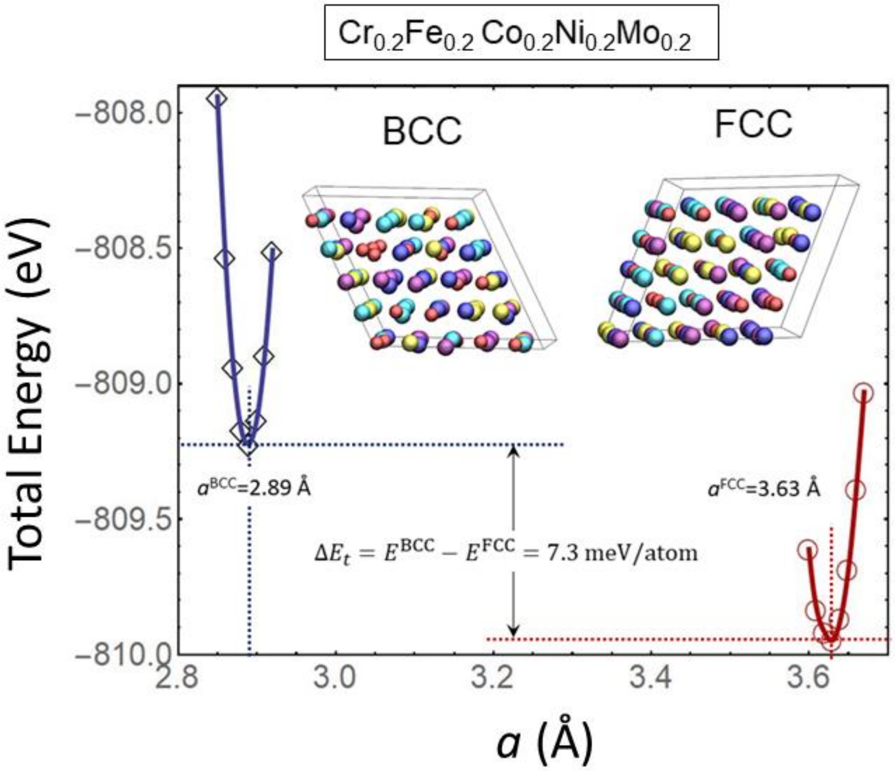

Supplementary Figure 4 | DFT simulation. Phase stability calculations by DFT simulation for a $Cr_{0.2}Fe_{0.2}Co_{0.2}Ni_{0.2}Mo_{0.2}$ HEA NP and its lattice constants (BCC: 2.89 Å; FCC: 3.63 Å).

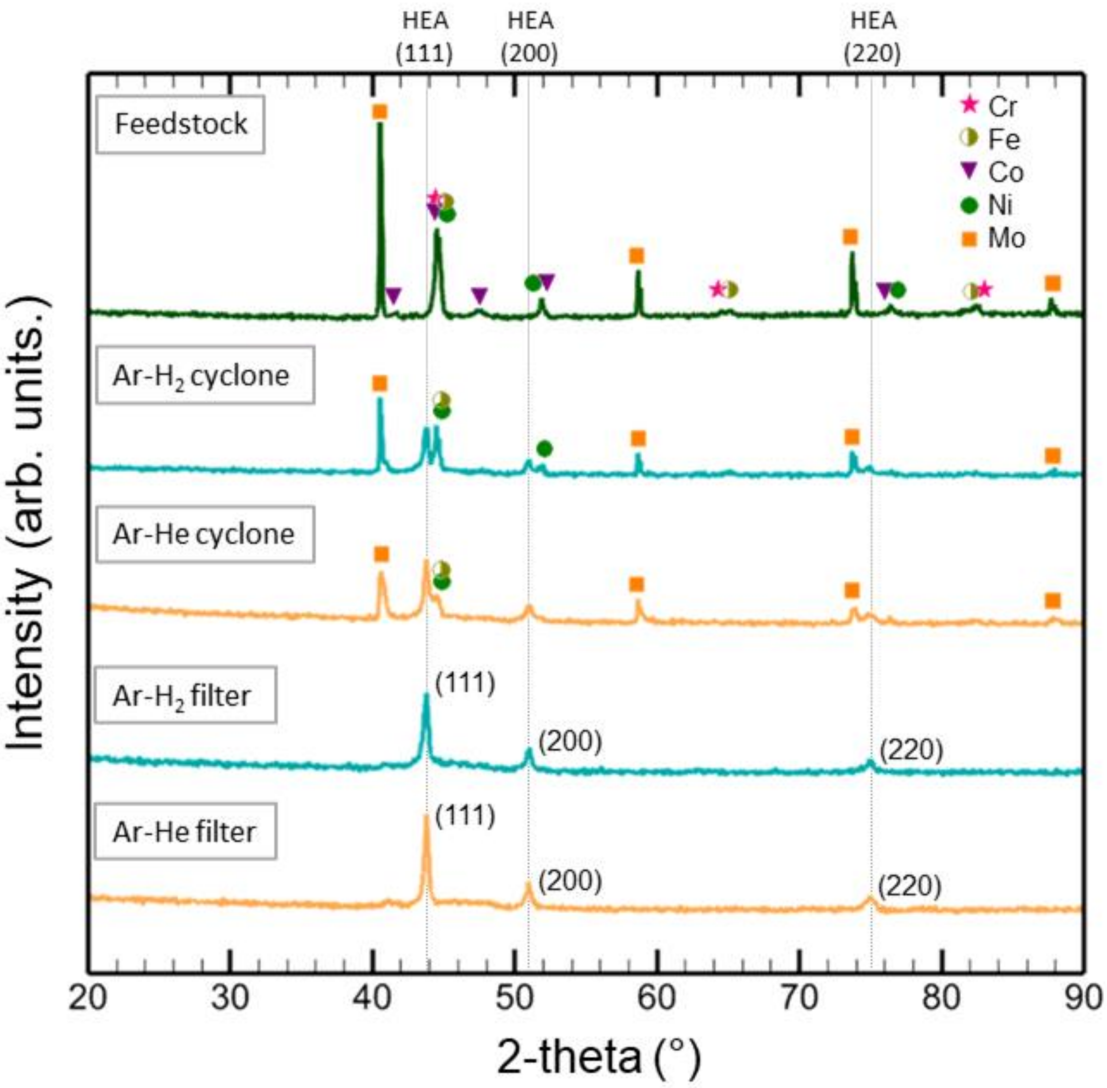

Supplementary Figure 5 | XRD analysis. XRD patterns of the feedstock mixture (Cr:Fe:Co:Ni:Mo = 1:1:1:1:1) and the HEA NP samples collected from different locations of the cyclone separator and the filter unit.

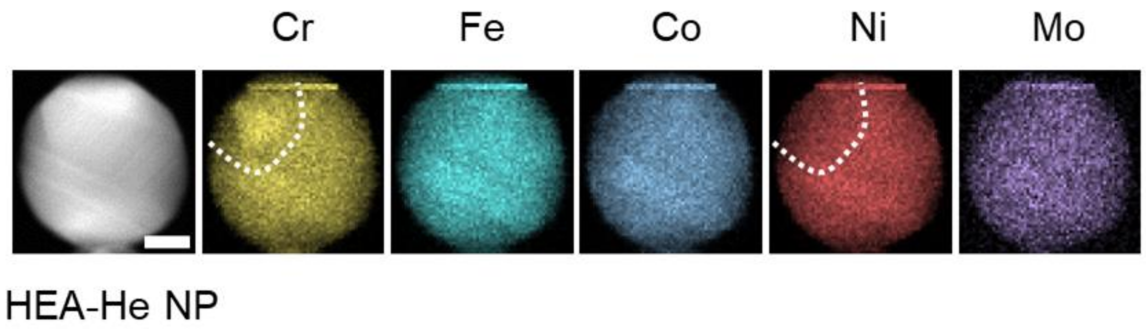

Supplementary Figure 6 | EDX elemental mapping. EDX elemental maps of a HEA NP produced with Ar-He plasma gas (HEA-He, He: 77.4%), showing a slight phase segregation in the particle. Scale bar, 25 nm.

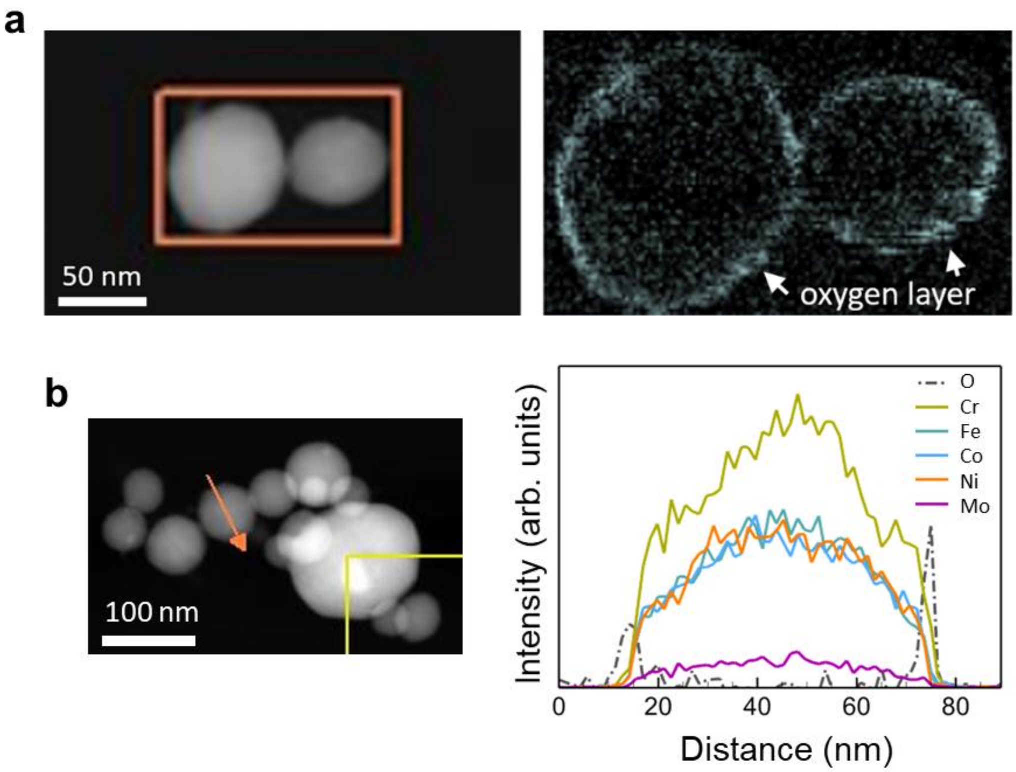

Supplementary Figure 7 | EELS and EDX mapping. a EELS elemental map (oxygen) of as-produced HEA NPs. Scale bar, 50 nm. **b** EDX line scan along the orange line across a HEA NP. Scale bar, 100 nm. Both analyses indicate the formation of oxygen layers at the surface; however, the oxygen concentration inside HEA NPs is negligible.

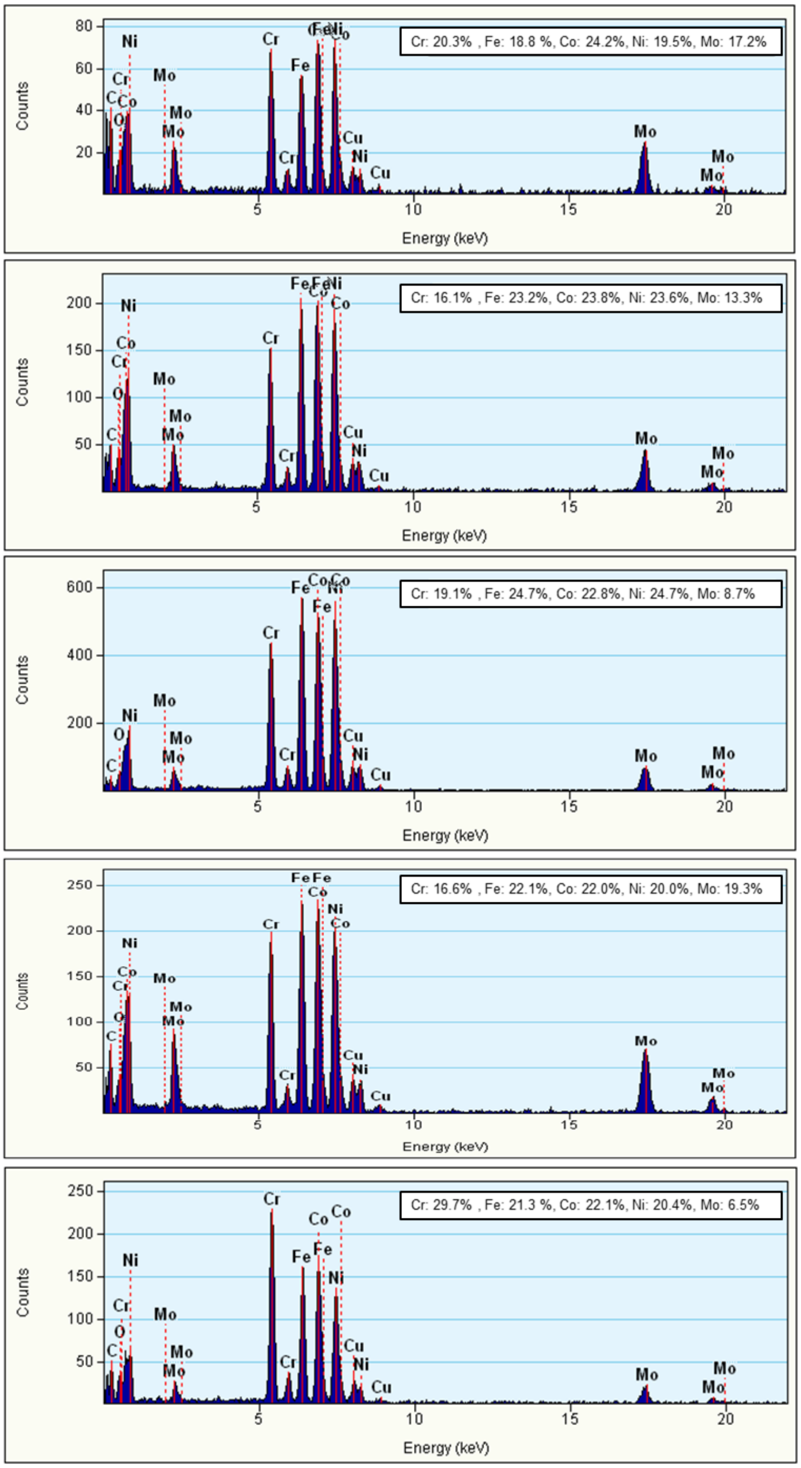

Supplementary Figure 8 | EDX analysis. Example EDX spectra of CrFeCoNiMo HEA NPs synthesized with Ar-H_2 (H_2: 8.3%) plasma gas.

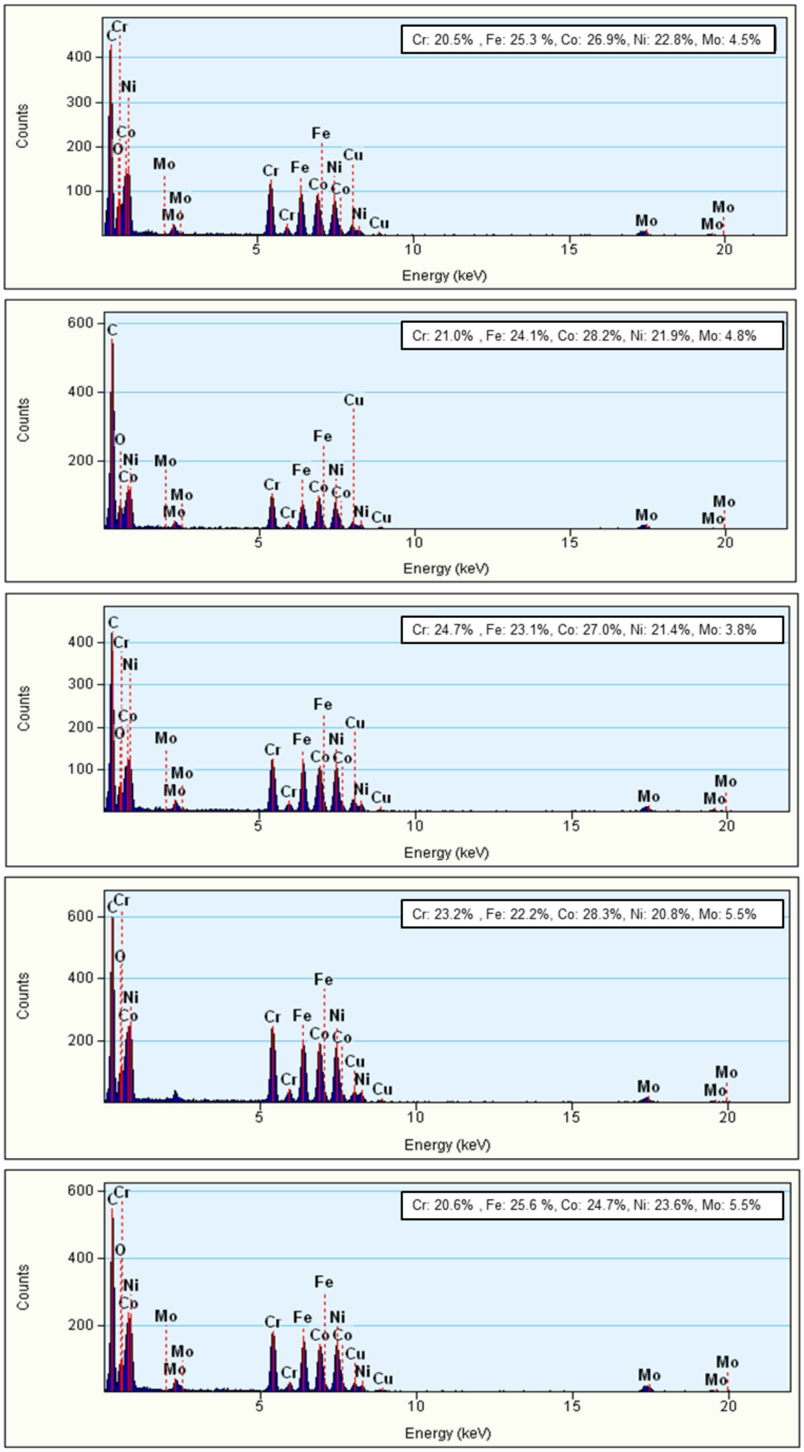

Supplementary Figure 9 | EDX analysis. Example EDX spectra of CrFeCoNiMo HEA NPs synthesized with Ar-He (He: 77.4%) plasma gas.

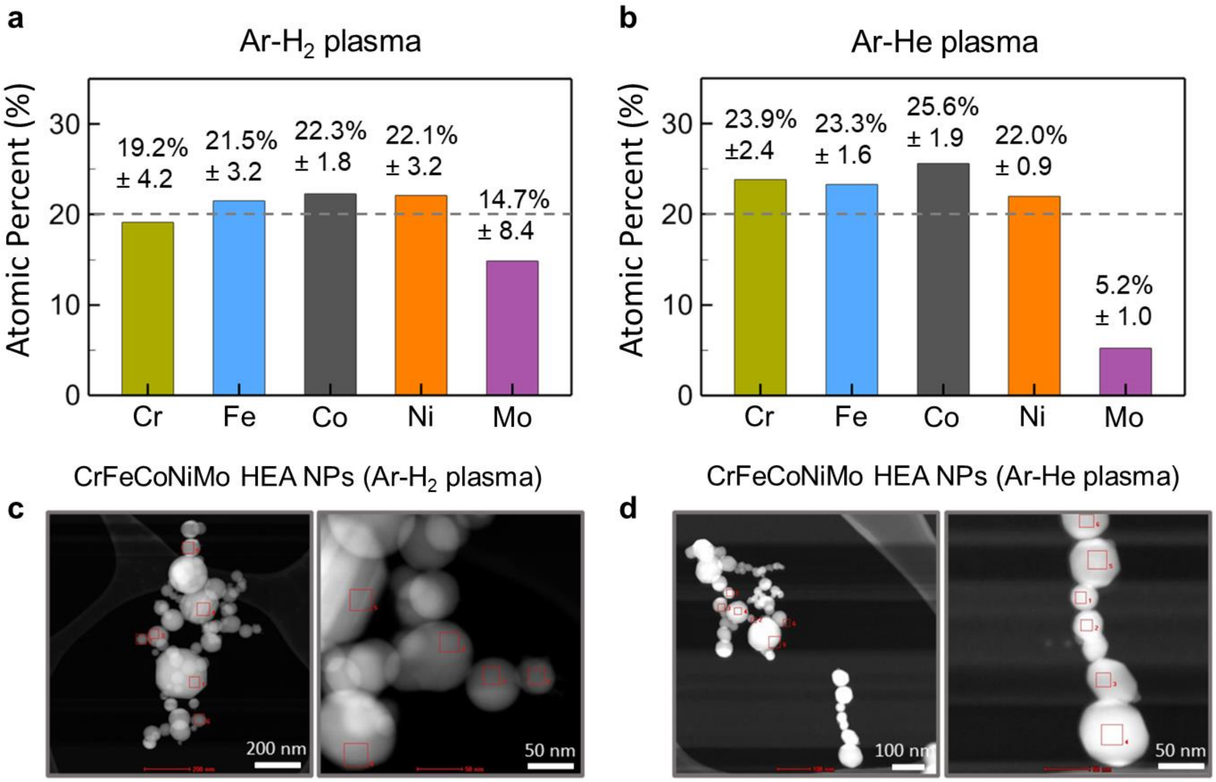

Supplementary Figure 10 | EDX analysis. Statistical analysis on composition of HEA NPs (CrFeCoNiMo) produced by **a** Ar-H_2 (H_2: 8.3%) plasma gas and **b** Ar-He (He: 77.4%) plasma gas. **c,d**, Corresponding HAADF-STEM images.

Supplementary Table 4 | Elemental ratio of CrFeCoNiMo HEA NP synthesized with different plasma gases of Ar-H_2 and Ar-He.

	Cr (%)	Fe (%)	Co (%)	Ni (%)	Mo (%)
Ar-H_2 plasma	19.2±4.23	21.5±3.19	22.3±1.75	22.1±3.16	14.7±8.35
Ar-He Plasma	23.9±2.36	23.3±1.6	25.6±1.89	22.0±0.85	5.2±0.94

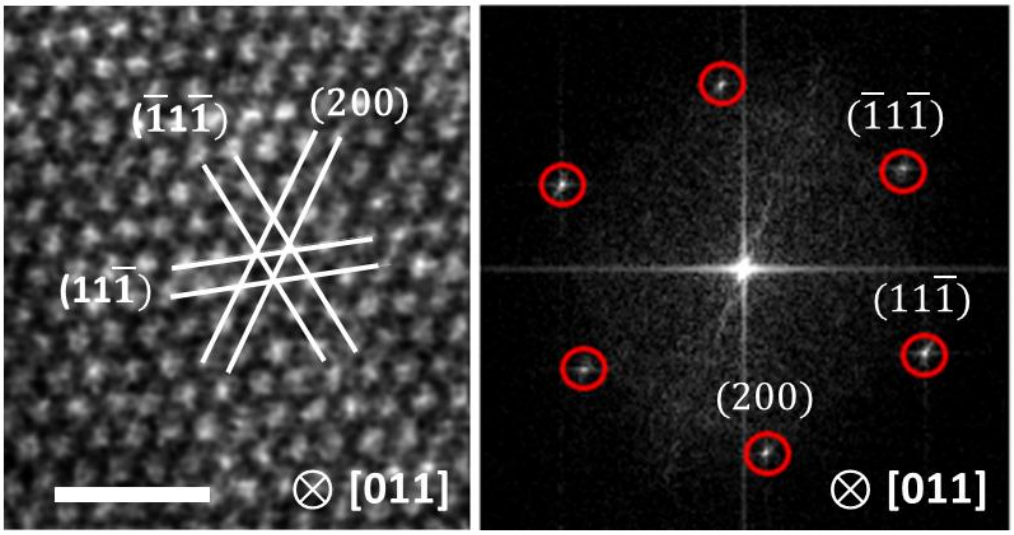

Supplementary Figure 11 | TEM analysis. Atomically-resolved HAADF-STEM image and the corresponding FFT analysis of a HEA NP. Scale bar, 1 nm.

Supplementary Table 5 | Physical properties of the metallic elements employed in this work (bulk).

Element	Atomic Number	Structure @RT	Structure @T_m	Atomic Radius (pm)	Pauling EN	VEC	T_m (K)	T_b (K)
Cr	24	BCC		124.91	1.66	6	2180	2944
Mn	25	BCC		135.00	1.55	7	1519	2334
Fe	26	BCC	BCC	124.12	1.83	8	1811	3134
Co	27	HCP	FCC	125.10	1.88	9	1768	3200
Ni	28	FCC		124.59	1.91	10	1728	3003
Cu	29	FCC		127.80	1.90	11	1358	2835
Mo	42	BCC		136.26	2.16	6	2896	4912

T_m: melting point, T_b: boiling point, RT: room temperature, EN: electronegativity, VEC: Valence electron concentration

Supplementary Note 1:

Thermal stability evaluation of HEA NPs

In order to study the thermal stability of the HEA NPs produced, we have annealed CrFeCoNiMo HEA NPs produced with H_2 and He plasmas (i.e., HEA-H_2 and HEA-He) at 1,173 K (900 °C) for 72 hours using a tube furnace with a continuous flow of argon.

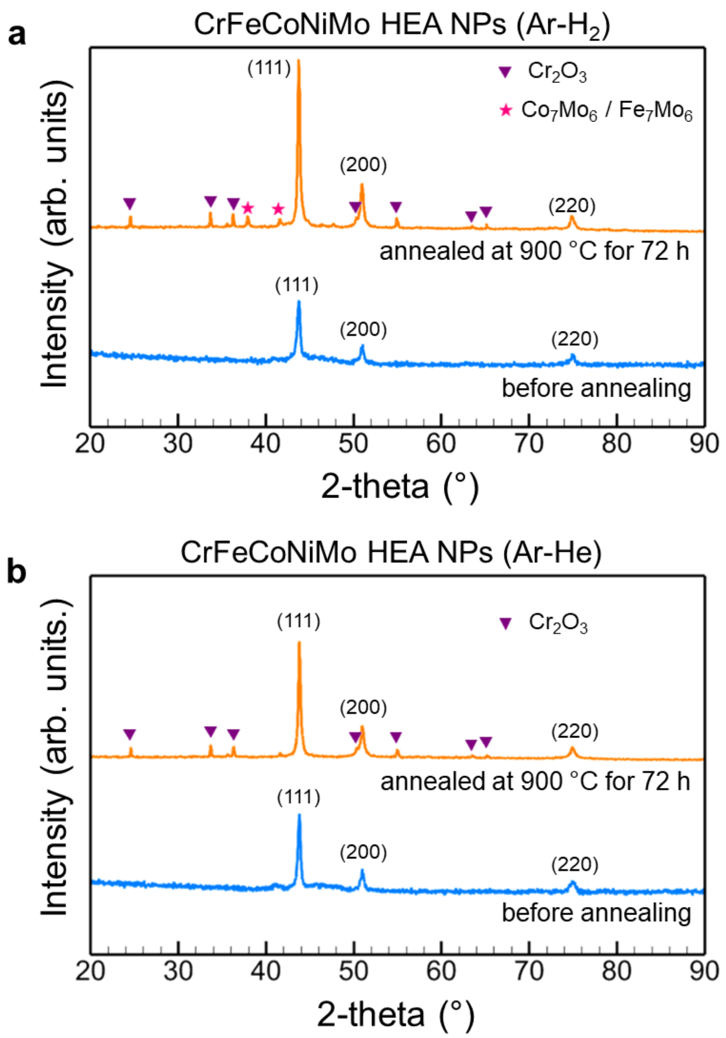

Supplementary Figure 12 | XRD analysis. XRD patterns of the HEA NPs after annealing at 1,173 K (900 °C) for 72 hours with a continuous flow of argon. **a** CrFeCoNiMo HEA NPs produced with hydrogen. **b** CrFeCoNiMo HEA NPs produced with helium.

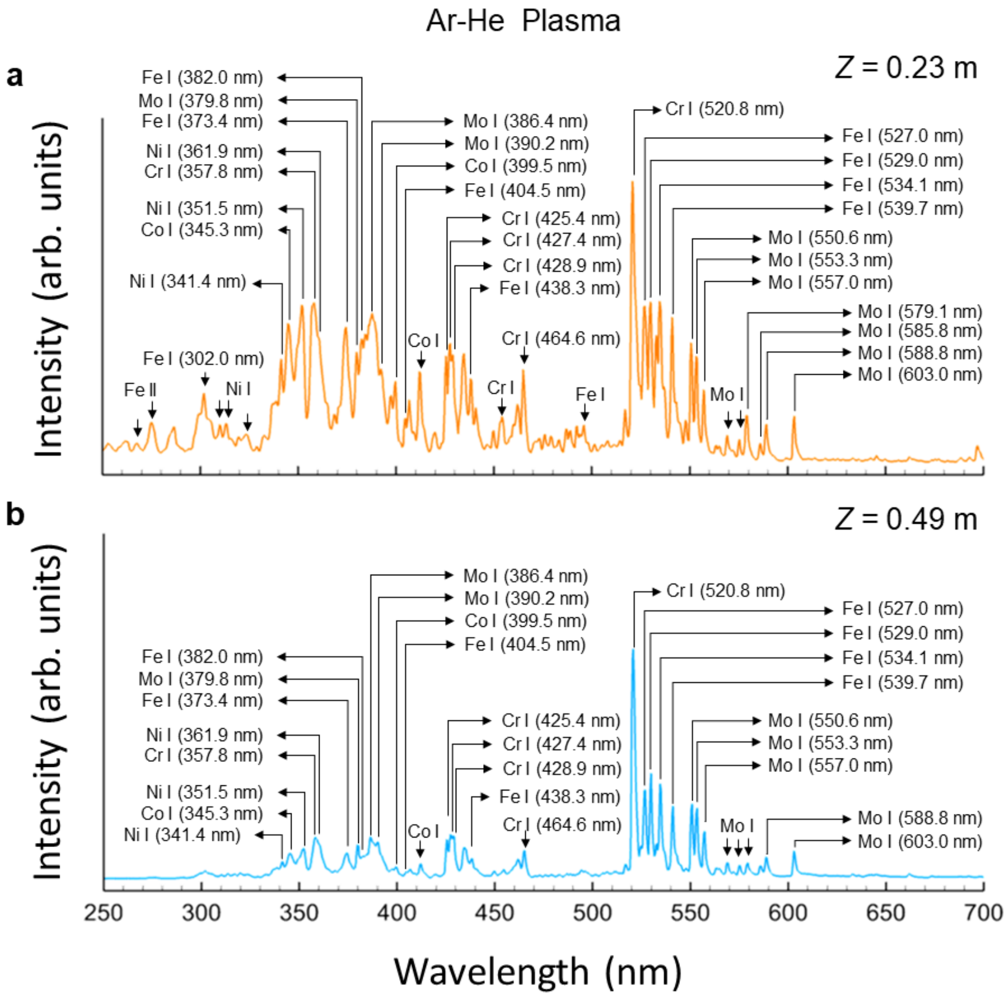

Supplementary Figure 13 | OES measurements. Optical emission spectra measured at **a** Z = 0.23 m and **b** Z = 0.49 m from the top of the plasma torch for the Ar-He (He: 77.4%) case.

Supplementary Note 2:

Homogenous nucleation temperature calculation

The homogeneous nucleation temperature of each metal was calculated using the self-consistent classical theory proposed in [1-3], where the nucleation rate J is given as follows:

$$J = \frac{\beta_{ij} n_s^2 S}{12} \sqrt{\frac{\Theta}{2\pi}} \exp\left[\Theta - \frac{4\Theta^3}{27(\ln S)^2}\right] \tag{1}$$

where β_{ij} is the collision frequency function between monomers and n_s is the equilibrium saturation monomer concentration at the temperature T. S and Θ are the supersaturation ratio and the dimensionless surface tension, respectively, which are defined as

$$S = \frac{P}{P_s}, \quad \Theta = \frac{\sigma s_1}{k_B T} \tag{2}$$

where P is the partial pressure of the metal species, P_s is the saturation vapor pressure at the temperature T, σ is the surface tension, s_1 is the surface of the monomer, and k_B is the Boltzmann constant. The collision frequency function between i-mers (a cluster containing i atoms) and j-mers (a cluster containing j atoms) is written as

$$\beta_{ij} = \left(\frac{3v_1}{4\pi}\right)^{\frac{1}{6}} \sqrt{\frac{6kT}{\rho_p}\left(\frac{1}{i}+\frac{1}{j}\right)} \left(i^{\frac{1}{3}} + j^{\frac{1}{3}}\right)^2 \tag{3}$$

Here v_1 is the volume of the monomer particle and ρ_p is the density of the monomer. As particles are nucleated from the monomer collisions, i and j are set to 1. It has been experimentally observed that the stable formation of particles occurs when the nucleation rate becomes higher than 1.0 cm^{-3} s^{-1}. The corresponding temperature is defined as the nucleation temperature in this work[3].

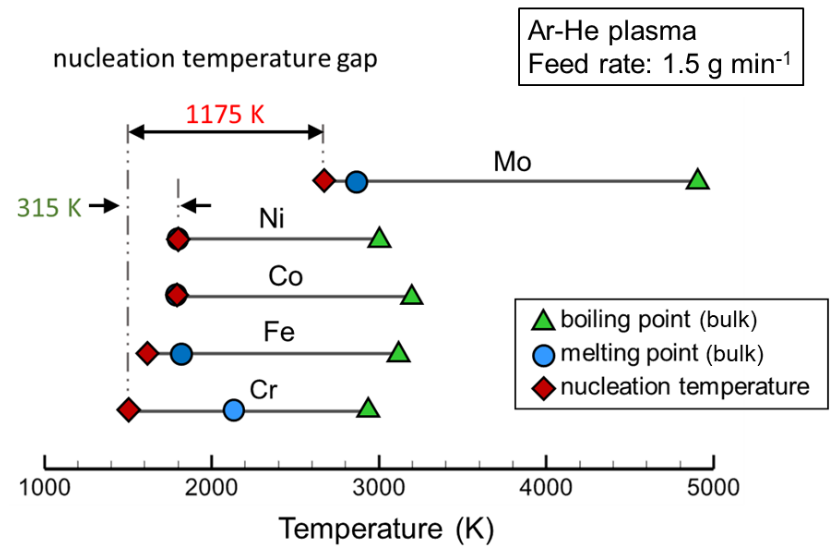

Supplementary Figure 14 | Nucleation temperature calculation. Nucleation temperatures calculated for each element in a vapor mix of Cr:Fe:Co:Ni:Mo = 1:1:1:1:1 produced at a feed rate of 1.5 g min^{-1}. Ar-He (He: 77.4%) case.

Supplementary Note 3:

Solidification temperature calculation

Supplementary Table 6 | Calculated solidification temperature of CrFeCoNiMo HEA NP.

	Homogeneous nucleation temperature (Mo)	Solidification temperature (HEA)	Average particle size (nm)
Ar-H_2 Plasma	2,655 K	1,865 K	60.8
Ar-He Plasma	2,669 K	1,919 K	39.7

The solidification temperature of a CrFeCoNiMo HEA NP was calculated with the following atomic ratios and diameters:

- Ar-H_2 case: $Cr_{0.19}Fe_{0.22}Co_{0.22}Ni_{0.22}Mo_{0.15}$; d = 60.8 nm.
- Ar-He case: $Cr_{0.24}Fe_{0.23}Co_{0.26}Ni_{0.22}Mo_{0.05}$; d = 39.7 nm.

It was assumed that HEA NPs are solidified when they reach their melting points. The melting temperature of a HEA NP for a given composition and diameter was calculated considering the melting temperature depression due to the size reduction,

$$T_{mp} = T_{mp,bulk} \left(1 - \frac{\alpha}{d}\right) \quad (4)$$

and the mixing rule,

$$T_{mp,HEA} = \chi_{Cr}T_{mp,Cr} + \chi_{Fe}T_{mp,Fe} + \chi_{Co}T_{mp,Co} + \chi_{Ni}T_{mp,Ni} + \chi_{Mo}T_{mp,Mo} \quad (5)$$

where T_{mp} is the melting temperature of nanoparticles, $T_{mp,bulk}$ is the melting temperature of bulk, α is a parameter determined by the solid and liquid surface energies and the bulk melting enthalpy, and χ is the atomic ratio of element. The values α = 1.5, 2.46, 2.49, 1.56, and 1.06 nm were used for Cr, Fe, Co, Ni, and Mo, respectively.

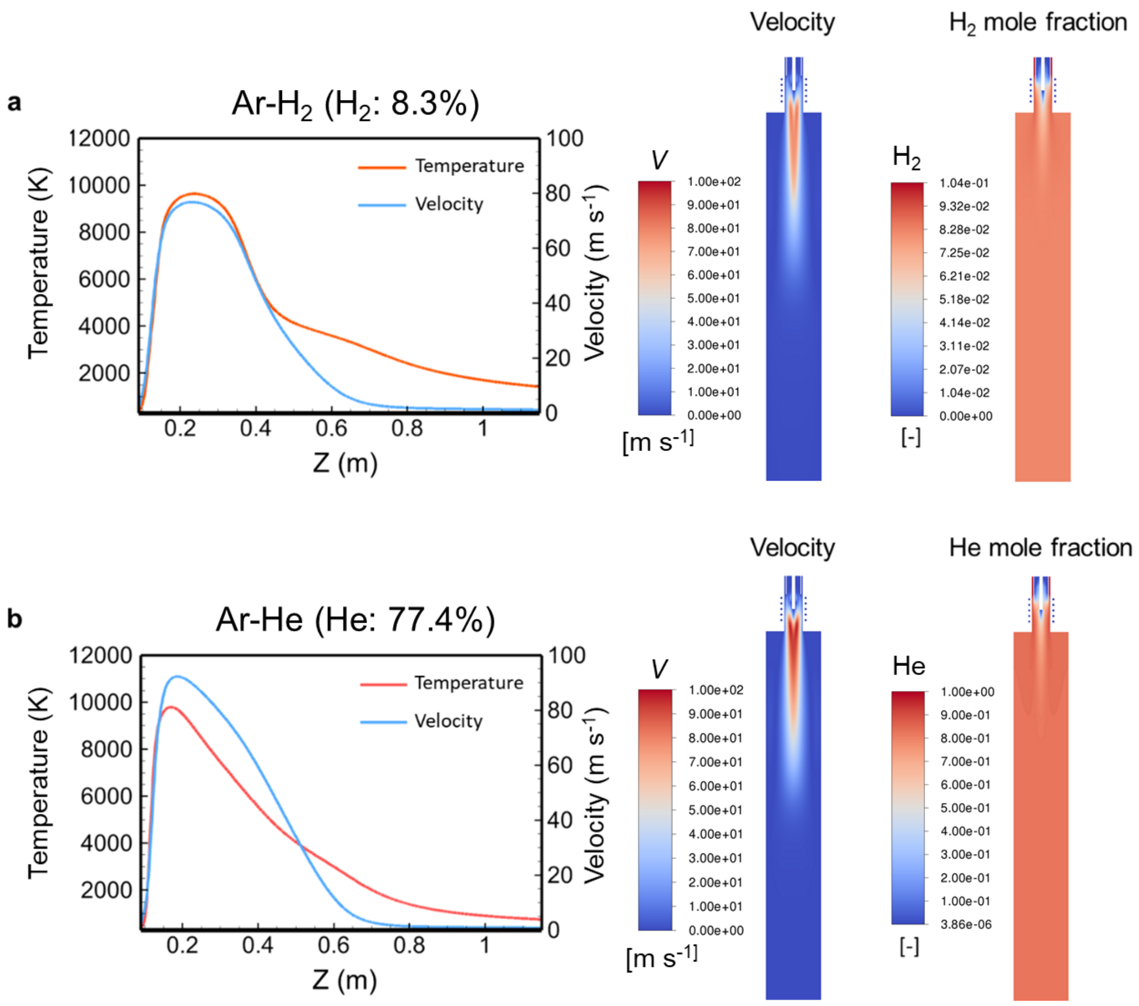

Supplementary Figure 15 | Thermofluid simulation. Calculated axial temperature and velocity profiles with corresponding velocity and mole fraction distributions. **a** Ar-H_2 (H_2: 8.3 %) plasma case. **b** Ar-He (He: 77.4 %) plasma case.

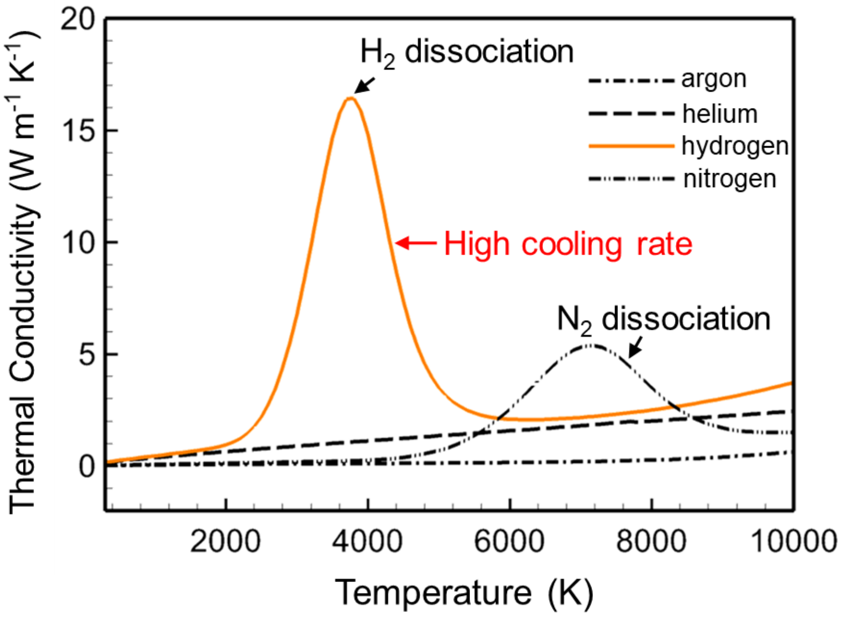

Supplementary Figure 16 | Thermal conductivities of gases. Thermal conductivity of various plasma gases of hydrogen, helium, nitrogen and argon for a temperature range of 300 K–10,000 K.

Supplementary Table 7 | The mixing enthalpy of binary elementary combinations[4].

	Co	Cr	Cu	Fe	Mn	Mo	Ni
Co	-	-4	6	-1	-5	-5	0
Cr	-4	-	12	-1	2	0	-7
Cu	6	12	-	13	4	19	4
Fe	-1	-1	13	-	0	-2	-2
Mn	-5	2	4	0	-	5	-8
Mo	-5	0	19	-2	5	-	-7
Ni	0	-7	4	-2	-8	-7	-

unit: kJ mol^{-1}

- **CrMnFeCoNi HEA-NPs**

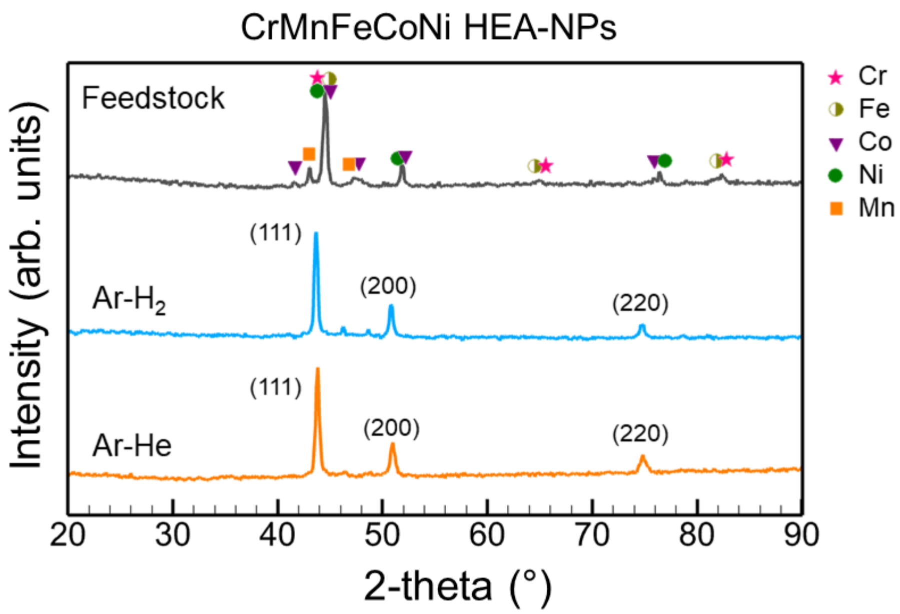

Supplementary Figure 17 | XRD analysis. XRD patterns of the feedstock mixture (Cr:Mn:Fe:Co:Ni = 1:1:1:1:1) and HEA NP samples collected from the filter unit.

Supplementary Table 8 | Interplanar spacing and lattice constants of CrMnFeCoNi HEA NP synthesized with Ar-H_2 and Ar-He plasma gases.

hkl	$h^2 + k^2 + l^2$	*d* spacing (Å)	Lattice constant (Å)
111	1.732	2.0701	3.5856
200	2	1.7957	3.5914
220	2.828	1.2709	3.5947
			Average: 3.5906

hkl	$h^2 + k^2 + l^2$	*d* spacing (Å)	Lattice constant (Å)
111	1.732	2.0657	3.5779
200	2	1.7892	3.5784
220	2.828	1.2681	3.5866
			Average: 3.5810

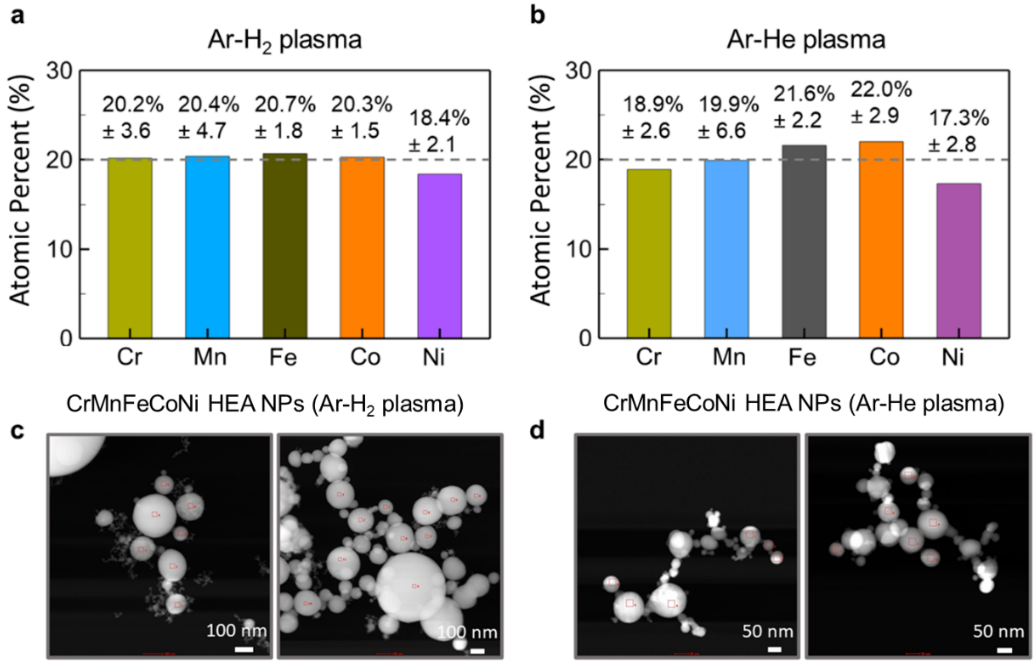

Supplementary Figure 18 | EDX analysis. Statistical analysis on composition of CrMnFeCoNi HEA NPs produced by **a** Ar-H_2 (H_2: 8.3%) plasma gas and **b** Ar-He (He: 77.4%) plasma gas. **c,d** Corresponding HAADF-STEM images.

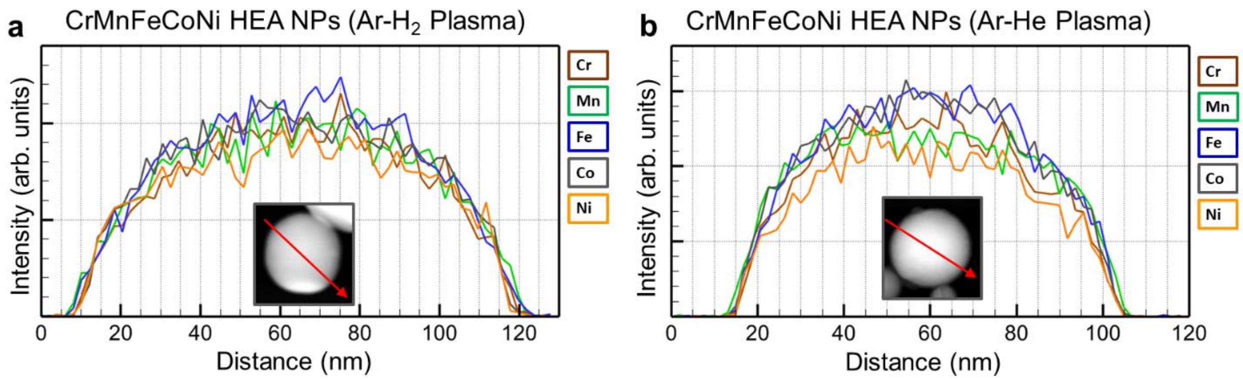

Supplementary Figure 19 | EDX line scans. EDX line scans of individual CrMnFeCoNi HEA NPs synthesized with **a** Ar-H_2 (H_2: 8.3%) plasma gas and **b** Ar-He (He: 77.4%) plasma gas.

- **MnFeCoNiCu HEA-NPs**

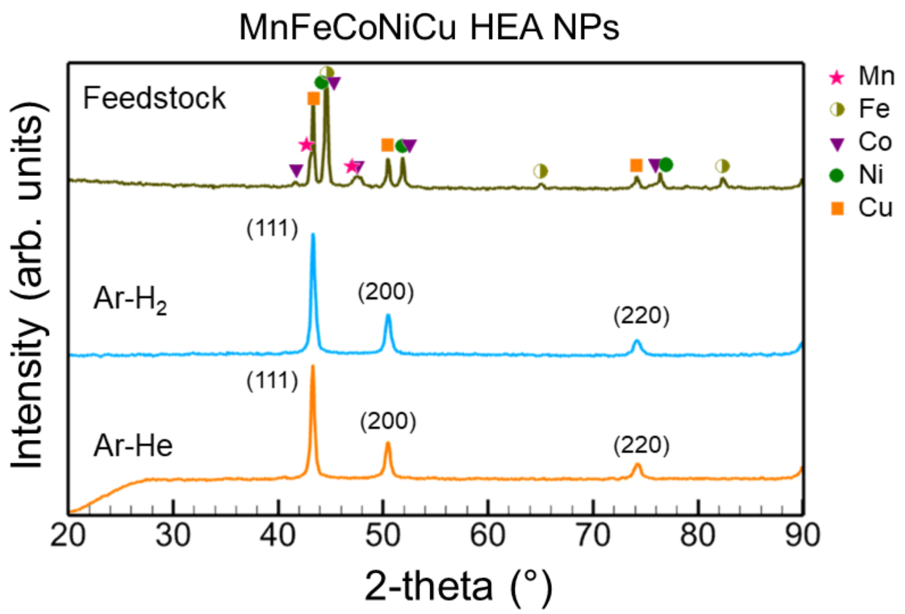

Supplementary Figure 20 | XRD analysis. XRD patterns of the feedstock mixture (Mn:Fe:Co:Ni:Cu = 1:1:1:1:1) and HEA NP samples collected from the filter unit.

Supplementary Table 9 | Interplanar spacing and lattice constants of MnFeCoNiCu HEA NP synthesized with Ar-H_2 and Ar-He plasma gases.

hkl	$h^2 + k^2 + l^2$	*d* spacing (Å)	Lattice constant (Å)
111	1.732	2.0881	3.6167
200	2	1.8055	3.6111
220	2.828	1.2767	3.6111
			Average: 3.6130

hkl	$h^2 + k^2 + l^2$	*d* spacing (Å)	Lattice constant (Å)
111	1.732	2.0881	3.6167
200	2	1.8088	3.6177
220	2.828	1.2767	3.6111
			Average: 3.6152

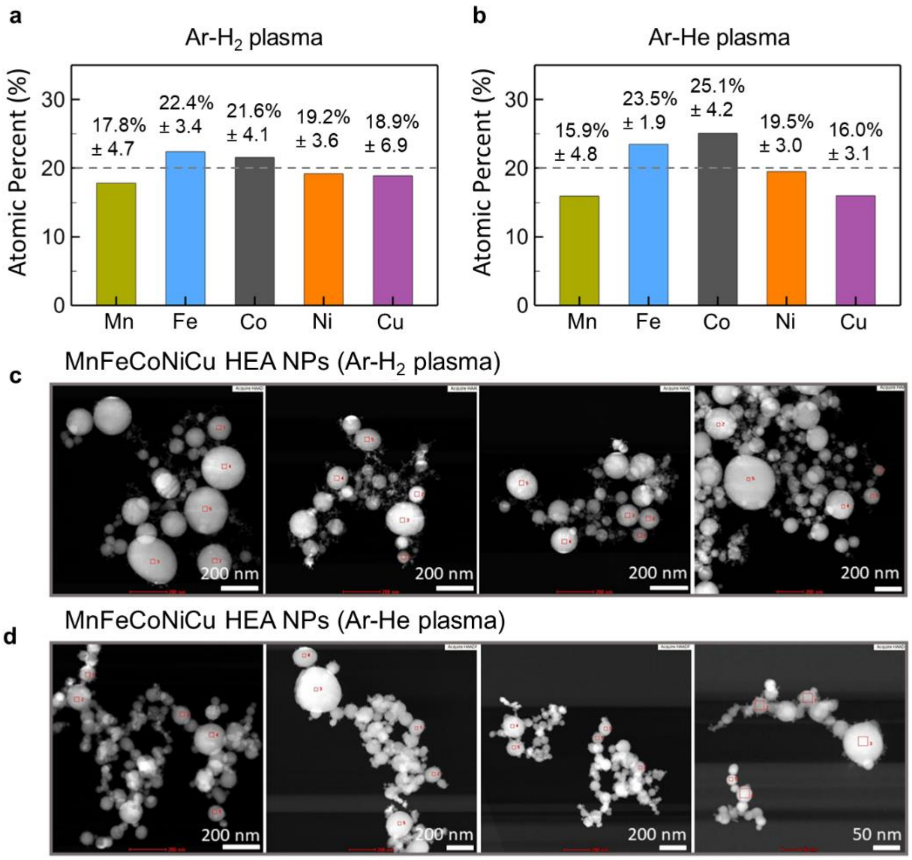

Supplementary Figure 21 | EDX analysis. Statistical analysis on composition of MnFeCoNiCu HEA NPs produced by **a** Ar-H_2 (H_2: 8.3%) plasma gas and **b** Ar-He (He: 77.4%) plasma gas. **c,d** Corresponding HAADF-STEM images.

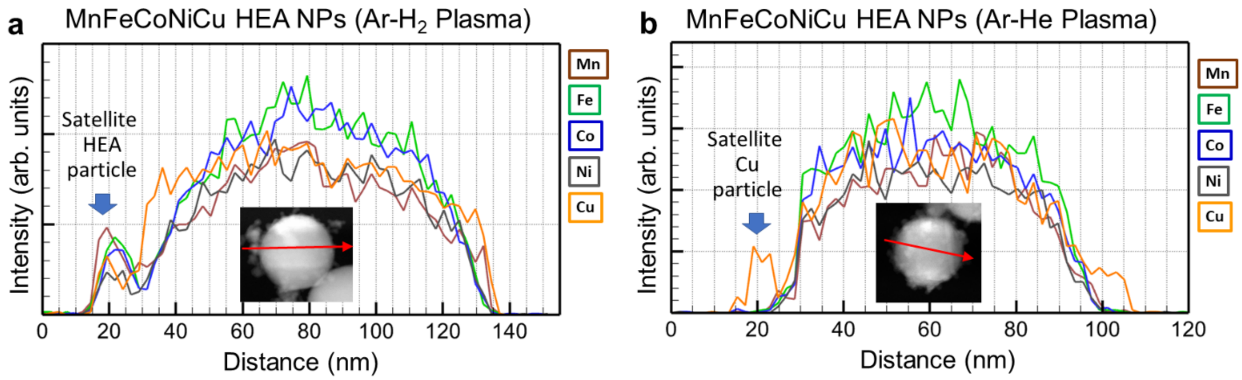

Supplementary Figure 22 | EDX line scans. EDX line scans of individual MnFeCoNiCu HEA NPs synthesized with **a** Ar-H_2 (H_2: 8.3%) plasma gas and **b** Ar-He (He: 77.4%) plasma gas.

- **CrFeCoNiCu HEA-NPs**

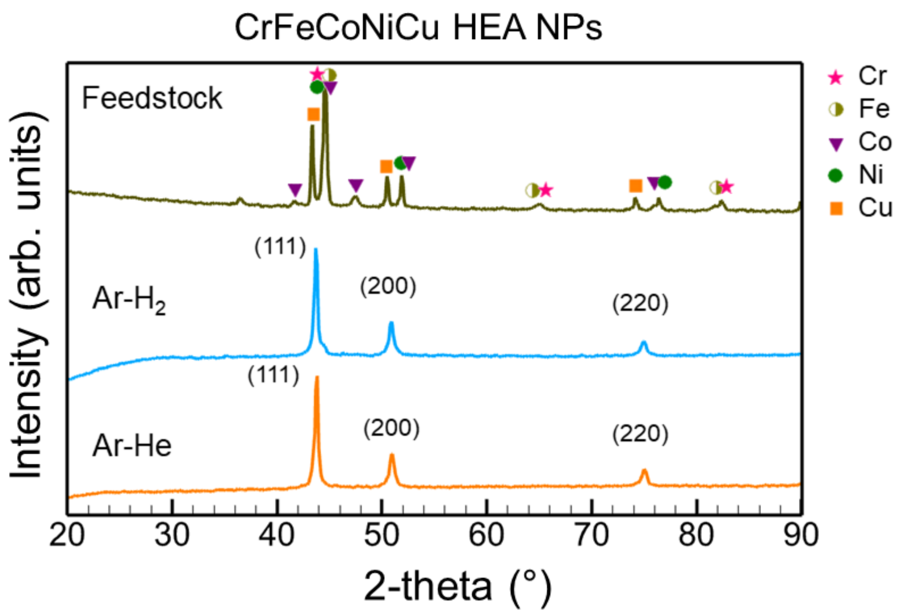

Supplementary Figure 23 | XRD analysis. XRD patterns of the feedstock mixture (Cr:Fe:Co:Ni:Cu = 1:1:1:1:1) and HEA NP samples collected from the filter unit.

Supplementary Table 10 | Interplanar spacing and lattice constants of CrFeCoNiCu HEA NP synthesized with Ar-H_2 Ar-He plasma gases.

hkl	$h^2 + k^2 + l^2$	*d* spacing (Å)	Lattice constant (Å)
111	1.732	2.0701	3.5856
200	2	1.7924	3.5849
220	2.828	1.2666	3.5826
			Average: 3.5843

hkl	$h^2 + k^2 + l^2$	*d* spacing (Å)	Lattice constant (Å)
111	1.732	2.0657	3.5779
200	2	1.7924	3.5849
220	2.828	1.2652	3.5785
			Average: 3.5804

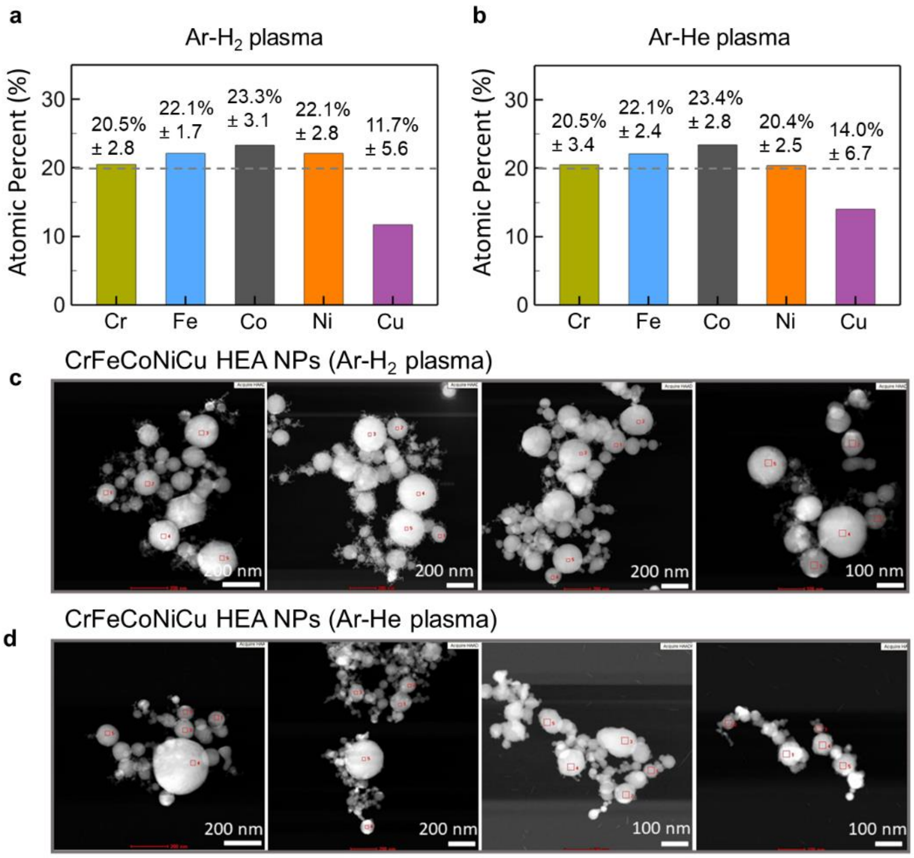

Supplementary Figure 24 | EDX analysis. Statistical analysis on composition of CrFeCoNiCu HEA NPs produced by **a** Ar-H_2 (H_2: 8.3%) plasma gas and **b** Ar-He (He: 77.4%) plasma gas. **c,d** Corresponding HAADF-STEM images.

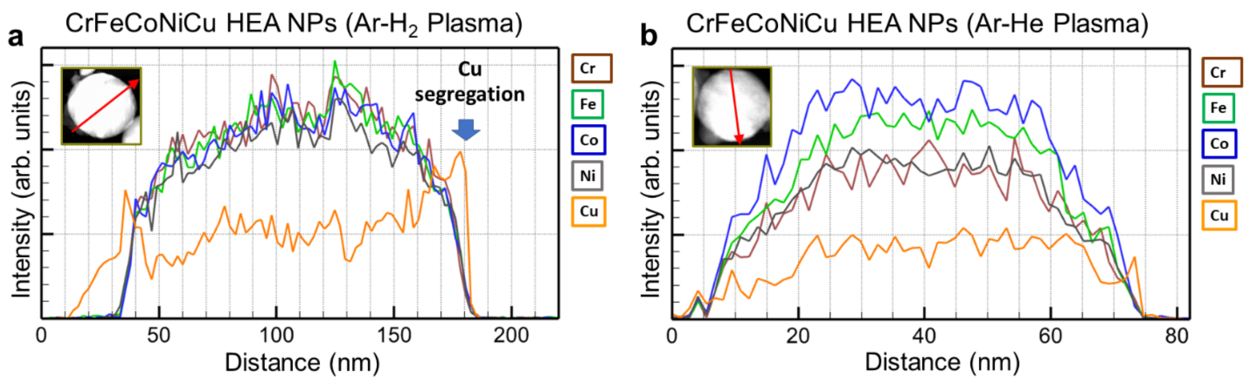

Supplementary Figure 25 | EDX line scans. EDX line scans of individual CrFeCoNiCu HEA NPs synthesized with **a** Ar-H_2 (H_2: 8.3%) plasma gas and **b** Ar-He (He: 77.4%) plasma gas.

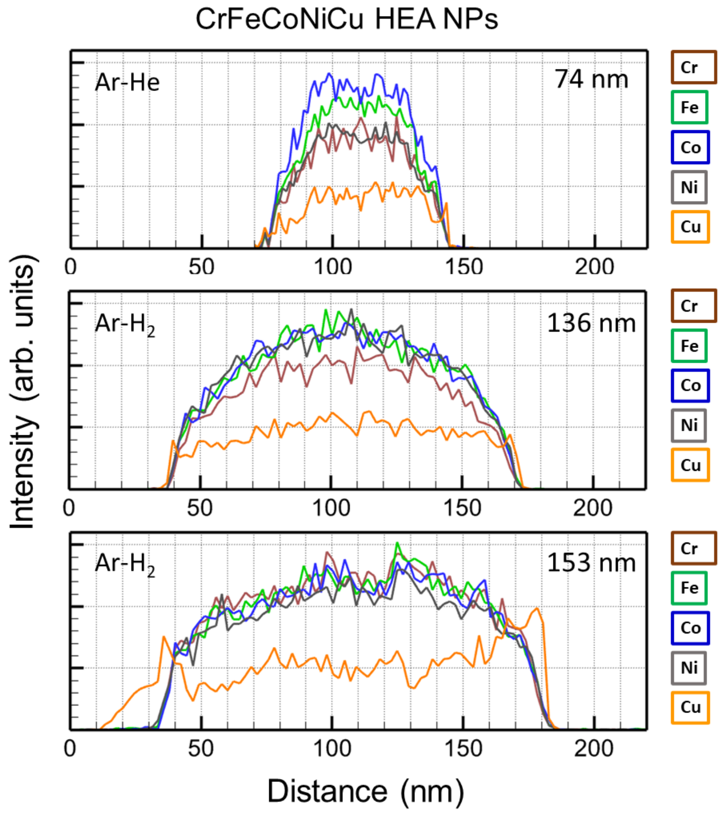

Supplementary Figure 26 | EDX line scans. EDX line scans of individual CrFeCoNiCu HEA NPs with different sizes.

Supplementary Table 11 | Summary of compositions of the HEA NPs produced in this work.

Ar-H_2 plasma	Cr (%)	Mn (%)	Fe (%)	Co (%)	Ni (%)	Cu (%)	Mo (%)
CrMnFeCoNi	20.2 ± 3.6	20.4 ± 4.7	20.7 ± 1.8	20.3 ± 1.5	18.4 ± 2.1		
MnFeCoNiCu		17.8 ± 4.7	22.4 ± 3.4	21.6 ± 4.1	19.2 ± 3.6	18.9 ± 6.9	
CrFeCoNiCu	20.5 ± 2.8		22.1 ± 1.7	23.3 ± 3.1	22.1 ± 2.8	11.7 ± 5.6	
CrFeCoNiMo	19.2 ± 4.2		21.5 ± 3.2	22.3 ± 1.8	22.1 ± 3.2		14.7 ± 8.4

Ar-He plasma	Cr (%)	Mn (%)	Fe (%)	Co (%)	Ni (%)	Cu (%)	Mo (%)
CrMnFeCoNi	18.9 ± 2.6	19.9 ± 6.6	21.6 ± 2.2	22.0 ± 2.9	17.3 ± 2.8		
MnFeCoNiCu		15.9 ± 4.8	23.5 ± 1.9	25.1 ± 4.2	19.5 ± 3.0	16.0 ± 3.1	
CrFeCoNiCu	20.5 ± 3.4		22.1 ± 2.4	23.4 ± 2.8	20.4 ± 2.5	14.0 ± 6.7	
CrFeCoNiMo	23.9 ± 2.4		23.3 ± 1.6	25.6 ± 1.9	22.0 ± 0.9		5.2 ± 1.0

Supplementary Note 4:

Thermofluid Simulation

• Plasma model

Vector potential:

$$\frac{\partial^2 A_\theta}{\partial z^2} + \frac{1}{r}\frac{\partial}{\partial r}(r\frac{\partial A_\theta}{\partial r}) - \frac{A_\theta}{r^2} = -\mu_0(J_{\text{coil}} + J_{\text{ind}}) \tag{6}$$

Electric and magnetic fields:

$$E_\theta = -i2\pi f A_\theta, \quad \mu_0 H_z = \frac{1}{r}\frac{\partial}{\partial r}(r\frac{\partial A_\theta}{\partial r}), \quad \mu_0 H_r = -\frac{\partial A_\theta}{\partial z} \tag{7}$$

Lorentz forces and Joule heating:

$$F_r = \frac{1}{2}\mu_0\sigma\, Re[E_\theta H_z^*], \;\; F_z = -\frac{1}{2}\mu_0\sigma\, Re[E_\theta H_r^*], \;\; P_{ohm} = \frac{1}{2}\sigma \text{Re}[E_\theta E_\theta^*] \tag{8}$$

• Thermofluid model

Mass conservation:

$$\frac{\partial(\rho u)}{\partial z} + \frac{1}{r}\frac{(\rho r v)}{\partial r} = S_p^c \tag{9}$$

Momentum conservation*:*

$$\rho(u\frac{\partial u}{\partial z} + v\frac{\partial u}{\partial r}) = -\frac{\partial p}{\partial z} + 2\frac{\partial}{\partial z}(\mu_{eff}\frac{\partial u}{\partial z}) + \frac{1}{r}\frac{\partial}{\partial r}\left[\mu_{eff} r\left(\frac{\partial u}{\partial r} + \frac{\partial v}{\partial z}\right)\right] + F_z + S_p^{mz}$$

$$\rho(u\frac{\partial v}{\partial z} + v\frac{\partial v}{\partial r}) = -\frac{\partial p}{\partial r} + \frac{2}{r}\frac{\partial}{\partial r}(\mu_{eff} r\frac{\partial v}{\partial r}) + \frac{1}{r}\frac{\partial}{\partial z}\left[\mu_{eff} r\left(\frac{\partial v}{\partial z} + \frac{\partial u}{\partial r}\right)\right] - \frac{2\mu_{eff} r}{r^2} + \frac{\rho w^2}{r} + F_r + S_p^{mr} \tag{10}$$

$$\rho(v\frac{\partial w}{\partial z} + u\frac{\partial w}{\partial r}) = \frac{\partial}{\partial z}(\mu_{eff}\frac{\partial w}{\partial z}) + \frac{1}{r}\frac{\partial}{\partial r}\left[\mu_{eff} r\frac{\partial w}{\partial r}\right] - \frac{w}{r}(\rho v + \frac{\mu_{eff}}{r} + \frac{\partial \mu_{eff}}{\partial r})$$

Energy conservation:

$$\rho(u\frac{\partial h}{\partial z}+v\frac{\partial h}{\partial r})=\frac{\partial}{\partial z}(\frac{\kappa_{eff}}{C_p}\frac{\partial h}{\partial z})+\frac{1}{r}\frac{\partial}{\partial r}\left[r\frac{\kappa_{eff}}{c_p}\frac{\partial h}{\partial r}\right]+P_{ohm}-R_{rad}+S_p^e \tag{11}$$

Turbulent kinetic energy:

$$\rho(u\frac{\partial K}{\partial z}+v\frac{\partial K}{\partial r})=\frac{\partial}{\partial z}((\mu_l+\frac{\mu_t}{\mathrm{Pr}_K})\frac{\partial K}{\partial z})+\frac{1}{r}\frac{\partial}{\partial r}\left[r(\mu_l+\frac{\mu_t}{\mathrm{Pr}_K})\frac{\partial K}{\partial r}\right]+G-\rho\varepsilon \tag{12}$$

Dissipation rate of turbulent kinetic energy:

$$\rho(u\frac{\partial \varepsilon}{\partial z}+v\frac{\partial \varepsilon}{\partial r})=\frac{\partial}{\partial z}((\mu_l+\frac{\mu_t}{\mathrm{Pr}_\varepsilon})\frac{\partial \varepsilon}{\partial z})+\frac{1}{r}\frac{\partial}{\partial r}\left[r(\mu_l+\frac{\mu_t}{\mathrm{Pr}_\varepsilon})\frac{\partial \varepsilon}{\partial r}\right]+\frac{\varepsilon}{K}(C_1G-C_2\rho\varepsilon) \tag{13}$$

Species conservation:

$$\rho(u\frac{\partial Y_k}{\partial z}+v\frac{\partial Y_k}{\partial r})=\frac{\partial}{\partial z}(\rho D_k\frac{\partial Y_k}{\partial z})+\frac{1}{r}\frac{\partial}{\partial r}\left[r(\rho D_k+\frac{\mu_t}{Sc_t})\frac{\partial Y_k}{\partial r}\right]+S_p^k \tag{14}$$

In the above equation, terms (S_p^c, S_p^e, S_p^{mr}, S_p^{mz}) related to particles were set to zero because the injection of metal powders was not considered.

The effective viscosity and thermal conductivity in the above equations include both laminar and turbulent components,

$$\mu_{eff}=\mu_l+\mu_t \text{ and } \kappa_{eff}=\kappa_l+\kappa_t \tag{15}$$

In the above equations, the turbulent viscosity μ_t and the turbulent thermal conductivity κ_t are defined as

$$\mu_t=\rho C_\mu\frac{K^2}{\varepsilon} \text{ and } \kappa_t=\frac{\mu_t C_p}{\mathrm{Pr}_t} \tag{16}$$

- **Computation domain and boundary conditions**

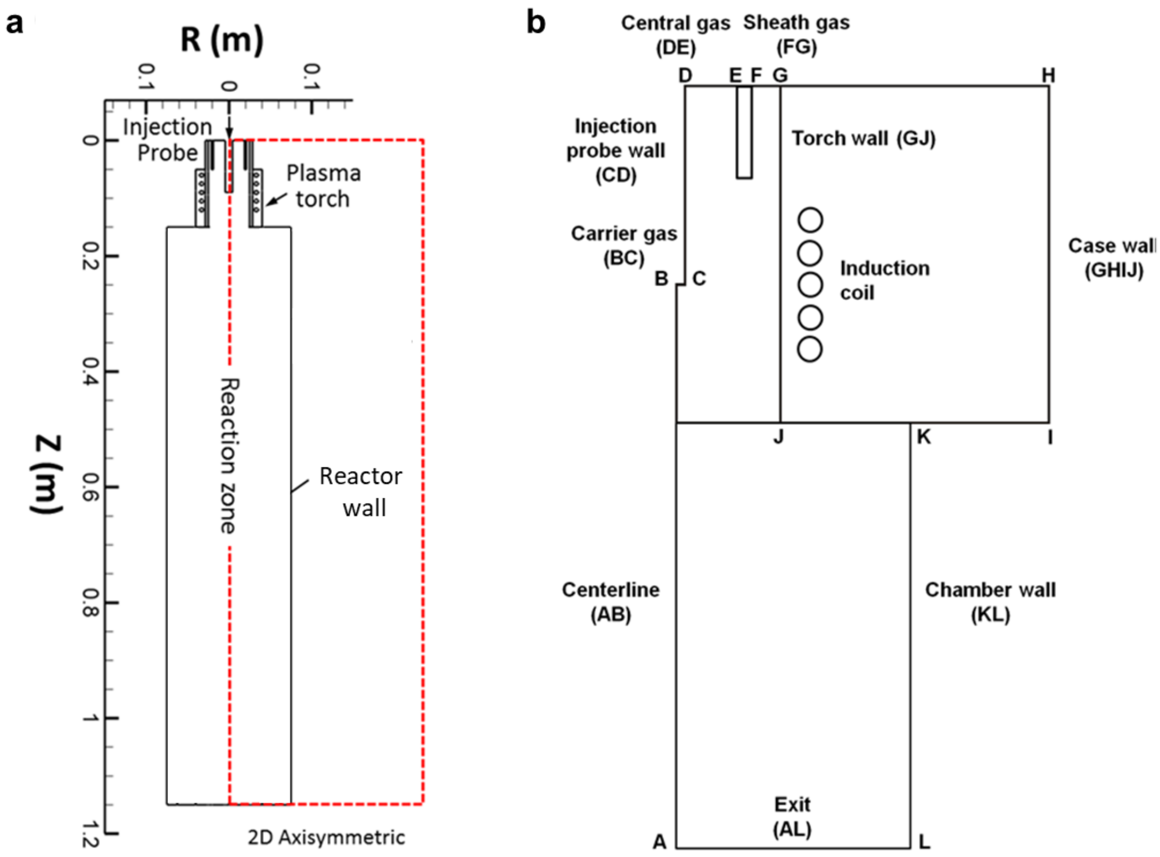

Supplementary Figure 27 | Computational geometry. **a** Computational domain. **b** Boundary.

Supplementary Table 12 | Boundary conditions for 2D numerical simulations.

Main Variables	Centerline AB	Probe wall CD	Tube wall EF	Torch wall GH	Case wall GHIJ
T	$\frac{\partial T}{\partial n} = 0$	$T = 300$ K	$T = 300$ K	$T = 300$ K	$T = 300$ K
$\mathbf{u}$	$\frac{\partial u}{\partial n} = 0$	$\mathbf{u} = 0$	-	$\mathbf{u} = 0$	$\mathbf{u} = 0$
K	$\frac{\partial K}{\partial n} = 0$	wall function	-	wall function	wall function
ε	$\frac{\partial \varepsilon}{\partial n} = 0$	wall function	-	wall function	wall function
Y	$\frac{\partial Y}{\partial n} = 0$	$\frac{\partial Y}{\partial n} = 0$	$\frac{\partial Y}{\partial n} = 0$	$\frac{\partial Y}{\partial n} = 0$	$\frac{\partial Y}{\partial n} = 0$
A_θ	$\frac{\partial A_\theta}{\partial n} = 0$	$A_\theta = 0$	$A_\theta = 0$	-	$A_\theta = 0$

Main Variables	Carrier gas BC	Central gas DE	Sheath gas FG	Chamber wall KL	Exit AL
T	$T = 300$ K	$T = 300$ K	$T = 300$ K	$T = 300$ K	$\frac{\partial T}{\partial z} = 0$
$\mathbf{u}$	$u = \frac{\text{flow rate}}{\text{area}}$	$u = \frac{\text{flow rate}}{\text{area}}$	$u = \frac{\text{flow rate}}{\text{area}}$	$\mathbf{u} = 0$	$\frac{\partial u}{\partial z} = 0$
K	$K = 0.005u^2$	$K = 0.005u^2$	$K = 0.005u^2$	wall function	$\frac{\partial K}{\partial z} = 0$
ε	$\varepsilon = 0.09\rho K^2/\mu$	$\varepsilon = 0.09\rho K^2/\mu$	$\varepsilon = 0.09\rho K^2/\mu$	wall function	$\frac{\partial \varepsilon}{\partial z} = 0$
Y	Ar-100%	Ar-100%	Ar-100%<br>Ar-H_2 (H_2: 10%)<br>He-100 %	$\frac{\partial Y}{\partial n} = 0$	$\frac{\partial Y}{\partial z} = 0$
A_θ	$A_\theta = 0$	$A_\theta = 0$	$A_\theta = 0$	$A_\theta = 0$	$A_\theta = 0$

Supplementary Nomenclature

A_θ	tangential component of magnetic vector potential (T m)
C_p	specific heat of fluid at constant pressure (J kg^{-1} K^{-1})
C_μ	constant in turbulence model
C_1	constant in turbulence model
C_2	constant in turbulence model
D_k	binary diffusion coefficient of species k (m^2 s^{-1})
E_θ	tangential component of electric field (V m^{-1})
F_r	radial component of Lorentz force (N m^{-3})
F_z	axial component of Lorentz force (N m^{-3})
f	frequency applied to RF induction coil (Hz)
G	product of the turbulent viscosity and viscous dissipation terms (kg m^{-1} s^{-3})
h	specific enthalpy of fluid (J kg^{-1})
h_c	heat transfer coefficient of fluid (W m^{-2} K^{-1})
H_r	radial component of magnetic field intensity (A m^{-1})
H_z	axial component of magnetic field intensity (A m^{-1})
$\mathrm{J_{coil}}$	coil current density (A m^{-2})
$\mathrm{J_{ind}}$	induced current density (A m^{-2})
K	turbulent kinetic energy (m^2 s^{-2})
P_{ohm}	heat generation by ohmic heating (W m^{-3})
p	static pressure of fluid (Pa)
Pr	Prandtl number of fluid
Pr_ε	constant in turbulence model
Pr_K	constant in turbulence model
Pr_t	turbulent Prandtl number
Re	particle Reynolds number
R_{rad}	radiational loss taken into account by using net emission coefficient (W m^{-3})
Sc_t	turbulent Schmidt number
S_p^c	mass generation through evaporation of particle injected (kg m^{-3} s^{-1})
S_p^e	heat exchange with particle injected (W m^{-3})
S_p^k	mass generation of kth species through evaporation of particle injected (kg m^{-3} s^{-1})
S_p^{mr}	radial momentum exchange with particle injected (N m^{-3})
S_p^{mz}	axial momentum exchange with particle injected (N m^{-3})
T	temperature of fluid (K)
$\mathbf{u}$	velocity of fluid (m s^{-1})
u	axial velocity of fluid (m s^{-1})
v	radial velocity of fluid (m s^{-1})
w	swirl velocity of fluid (m s^{-1})
Y_k	mass fraction of species k

Greek

ε dissipation rate of turbulent kinetic energy ($m^2 s^{-3}$)

κ_l laminar thermal conductivity of fluid ($W m^{-1} K^{-1}$)

Supplementary References

1. Girshick, S. L. & Chiu, C. P. Kinetic nucleation theory: A new expression for the rate of homogeneous nucleation from an ideal supersaturated vapor. *J. Chem. Phys*. **93**, 1273-1277 (1990).
2. Girshick, S. L., Chiu, C. P. & McMurry, P. H. Time-dependent aerosol models and homogeneous nucleation rates. *Aerosol Sci. Technol*. **13**, 465-477 (1990).
3. Friedlander S. K. *Smoke, Dust, and Haze: Fundamentals of Aerosol Dynamics* 2nd ed., (Oxford University Press, Oxford, U.K., 2000).
4. Takeuchi, A. & Inoue, A. Calculations of mixing enthalpy and mismatch entropy for ternary amorphous alloys. *Materials transactions, JIM*, **41**, 372-1378 (2000).
